# Hypertrophic Cardiomyopathy and the Risk of Bradyarrhythmia and Permanent Pacemaker Implantation: A Nationwide Cohort Study

**DOI:** 10.1002/clc.70454

**Published:** 2026-08-25

**Authors:** Tsu‐An Yang, Yu‐Yu Hsiao, Shang‐Ju Wu, Yu‐Shan Chien, Ming‐Jen Kuo, Yu‐Shan Huang, Guan‐Yi Li, Ching‐Heng Lin, Cheng‐Hung Li, Jiunn‐Cherng Lin, Jin‐Long Huang, Yun‐Yu Chen, Li‐Wei Lo, Yenn‐Jiang Lin, Yu‐Cheng Hsieh, Shih‐Ann Chen

**Affiliations:** ^1^ Department of Family Medicine Changhua Christian Hospital Changhua Taiwan; ^2^ Cardiovascular Center and Department of Internal Medicine Taichung Veterans General Hospital Taichung Taiwan; ^3^ Department of Internal Medicine, Faculty of Medicine, Institute of Clinical Medicine National Yang Ming Chiao Tung University School of Medicine Taipei Taiwan; ^4^ Department of Data Science and Big Data Analytics, and Department of Financial Engineering Providence University Taichung Taiwan; ^5^ Department of Medical Research Taichung Veterans General Hospital Taichung Taiwan; ^6^ Department of Post‐Baccalaureate Medicine, Cardiovascular Research Center College of Medicine, National Chung Hsing University Taichung Taiwan; ^7^ Department of Telemedicine Center Taichung Veterans General Hospital Taichung Taiwan; ^8^ Department of Medical Education Taichung Veterans General Hospital Taichung Taiwan; ^9^ Heart Rhythm Center and Division of Cardiology, Department of Medicine Taipei Veterans General Hospital Taipei Taiwan; ^10^ China Medical University Hospital Taichung Taiwan

**Keywords:** atrial fibrillation, bradyarrhythmia, hypertrophic cardiomyopathy, permanent pacemaker, sick sinus syndrome

## Abstract

**Background:**

Hypertrophic cardiomyopathy (HCM) is associated with various arrhythmias, including bradyarrhythmia. The relationship between HCM and bradyarrhythmia, and subsequent need for permanent pacemaker (PPM) implantation, remains unclear. This study aimed to evaluate the risk of bradyarrhythmia and PPM implantation in HCM patients.

**Methods:**

This retrospective, population‐based cohort study identified patients with newly diagnosed HCM from 2012 to 2017 in Taiwan's National Health Insurance Research Database. Patients with prior bradyarrhythmia or PPM implantation were excluded. A control cohort was selected by matching patients without HCM to the study cohort by age and gender in a 1:1 ratio. The primary and secondary outcomes were the occurrence of bradyarrhythmia and PPM implantation, respectively. A multivariate Cox proportional hazards regression model was used to evaluate the hazard ratios (HR) for the outcomes.

**Results:**

A total of 15 266 patients with a mean age of 63.8 ± 14.5 years constituted the HCM (*n* = 7633) and control (*n* = 7633) cohorts. During a follow‐up period of 4.05 ± 2.03 years, HCM was associated with higher risks of bradyarrhythmia (adjusted HR: 2.15, *p* < 0.001) and PPM implantation (adjusted HR: 6.58, *p* < 0.001). In patients with HCM, the presence of AF increased the risks of both bradyarrhythmia (adjusted HR: 1.61, *p* < 0.001) and PPM implantation (adjusted HR: 2.12, *p* < 0.001), while HF increased the risks of PPM implantation (adjusted HR: 1.61, *p* < 0.001).

**Conclusions:**

Patients with HCM had higher risks of bradyarrhythmia and PPM implantation. Patients with HCM should be carefully monitored for AF and HF, which might increase the risks of future PPM implantation.

## Graphical abstract

Patients with hypertrophic cardiomyopathy had significantly higher risks of bradyarrhythmia and permanent pacemaker implantation than matched controls. Among patients with HCM, atrial fibrillation increased the risks of both outcomes, while heart failure was associated with a higher risk of permanent pacemaker implantation.

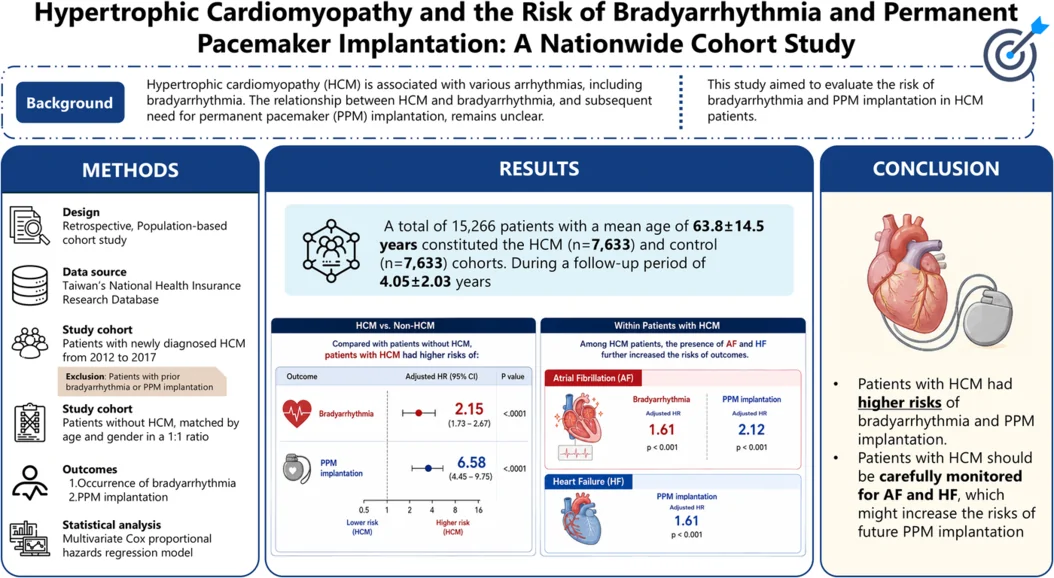

## 1 Introduction

Hypertrophic cardiomyopathy (HCM) is an inherited cardiac disorder caused by gene mutations that result in dysfunctional sarcomere proteins such as β‐myosin heavy chain and α‐tropomyosin. While ventricular tachyarrhythmias are a well‐documented cause of sudden cardiac death in HCM patients [1], bradyarrhythmias, which are less frequently studied, could also significantly impact patient outcomes and are therefore important [2, 3, 4, 5, 6]. In patients with HCM, scarred myocardium might impair both the conduction and propagation of electrical impulses [7]. The ischemic insult to the hypertrophied myocardium and conduction system might further exacerbate conduction disturbance and subsequently lead to bradycardia [8]. Therefore, understanding the progression from HCM to bradycardia and the subsequent need for pacemaker implantation is essential for improving patient management.

Previous studies investigating the future risks of bradycardia and pacemaker implantation in HCM have been limited by small sample sizes, restricting the generalizability of their findings [9]. In a cohort study that enrolled 161 patients with HCM, the odds of future bradyarrhythmic events were as high as 8% (13 patients), suggesting that HCM patients frequently experience bradyarrhythmias [10]. Additionally, a single‐center study analyzed 101 HCM patients referred for evaluation of arrhythmia‑related symptoms and found that 28.7% had primary bradyarrhythmia [2]. In another cohort of 451 patients with HCM, primary bradyarrhythmia was the indication for permanent pacemaker (PPM) implantation in 36 patients (8%) [9]. These limited sample sizes highlight the need for larger studies to better understand the risks and outcomes associated with bradycardia and pacemaker implantation in this population. The current study aimed to address this gap by enrolling a significantly larger cohort of patients and investigating the risks of bradyarrhythmia and PPM implantation in patients with HCM. The robust data reported herein might lead to improved clinical practice and outcomes in this patient group.

## 2 Materials and Methods

### 2.1 Research Database

The Taiwan National Health Insurance Research Database (NHIRD) contains comprehensive records of outpatient visits, hospital admissions, prescriptions, and disease diagnoses. The NHIRD currently covers 99.82% of the healthcare services used by Taiwan's 23 million residents which was established in 1995. Diagnoses in the database were registered according to the International Classification of Diseases, 9th and 10th editions, Clinical Modification (ICD‐9‐CM, ICD‐10‐CM). Details of ICD‐9‐CM and ICD‐10‐CM codes are shown in Supporting Information S1: Table 1. In this retrospective cohort study, we employed a systematic random sampling method using population data from 2012 to 2017. To ensure patient confidentiality, the National Health Research Institute (NHRI) encrypts all personal information before releasing the data for academic research. The study protocol was approved by the Institutional Review Board of Taichung Veterans General Hospital (CE25432B) and in accordance with the Declaration of Helsinki (Table 1).

**Table 1:** Baseline characteristics of the study and control groups.

Variable	Total (*n* = 15,266)		No HCM (*n* = 7633)		HCM (*n* = 7633)		*p* value
	*n*	(%)	*n*	(%)	*n*	(%)	
Age, years (mean ± SD)	63.8 ± 14.5		63.8 ± 14.4		63.8 ± 14.5		0.776^b^
Gender							0.883
Female	6614	(43.3)	3302	(43.3)	3312	(43.4)	
Male	8652	(56.7)	4331	(56.7)	4321	(56.6)	
Diabetes mellitus	3594	(23.5)	2040	(26.7)	1554	(20.4)	< 0.0001
Atrial fibrillation	1310	(8.6)	235	(3.1)	1075	(14.1)	< 0.0001
Heart failure	2120	(13.9)	418	(5.5)	1702	(22.3)	< 0.0001
Hypertension	8727	(57.2)	3735	(48.9)	4992	(65.4)	< 0.0001
Stroke or transient ischemic attack	2023	(13.3)	1020	(13.4)	1003	(13.1)	0.703
Vascular disease^a^	1027	(6.7)	424	(5.6)	603	(7.9)	< 0.0001
Hyperlipidemia	4370	(28.6)	1926	(25.2)	2444	(32.0)	< 0.0001
Ischemic heart disease	4378	(28.7)	1219	(16.0)	3159	(41.4)	< 0.0001
Peripheral vascular disease	372	(2.4)	164	(2.2)	208	(2.7)	0.024
Valvular heart disease	1911	(12.5)	323	(4.2)	1588	(20.8)	< 0.0001
Chronic obstructive pulmonary disease	1753	(11.5)	722	(9.5)	1031	(13.5)	< 0.0001
Renal disease	1332	(8.7)	583	(7.6)	749	(9.8)	< 0.0001
Medications							
Beta blocker	3868	(25.3)	1206	(15.8)	2662	(34.9)	< 0.0001
ACEI or ARB	2899	(19.0)	1099	(14.4)	1800	(23.6)	< 0.0001
ASA (Aspirin)	3307	(21.7)	1206	(15.8)	2101	(27.5)	< 0.0001

*Note:* Brady‐arrhythmia includes sick sinus syndrome, AV block, and junctional bradycardia.

a Vascular disease was defined as myocardial infarction or peripheral vascular disease;

^b^ *t* test; Chi‐squared test or Fisher's exact test for all other *p* values.

### 2.2 Study Population

We identified patients aged ≥ 18 years with a new diagnosis of HCM (ICD‐9‐CM code 425.1; ICD‐10‐CM codes I42.1, I42.2) from 2012 to 2017. The diagnosis of HCM was further validated if the patient had at least three outpatient visits within a year with a diagnostic code for HCM, or at least one hospitalization with the HCM diagnostic code. Cardiovascular comorbidities were identified based on the following ICD‐9‐CM and ICD‐10‐CM codes if the patient had at least one hospitalization or at least three consecutive outpatient visits for any of the listed diseases: AF, hypertension, stroke, hyperlipidemia, coronary artery disease (CAD), chronic obstructive pulmonary disease (COPD), congestive heart failure (CHF), valvular heart disease (VHD), peripheral vascular disease (PVD), and chronic kidney disease (CKD). Patients with a prior history of bradyarrhythmia or PPM implantation before the index medical visit were excluded from the analysis because these were the study outcomes. Patients who underwent septal myectomy and alcohol septal ablation were identified using the procedure codes 68010B and 68048B, respectively.

### 2.3 Study Outcomes

The primary outcome of this study was the occurrence of bradyarrhythmia, defined as a composite of sick sinus syndrome, atrioventricular block, and junctional bradycardia. The secondary outcome was the occurrence of PPM implantation, identified by procedure codes of 47103A, N20014, 68011B, 68012B, and 68041B in the NHIRD during the follow‐up period (2011−2018). The study endpoint was defined as any one of the events that occurred after patients' enrollment during the follow‐up period.

### 2.4 Statistical Analysis

Continuous variables are presented as mean ± standard deviation (SD), and categorical variables as proportions. Differences for continuous or categorical variables were evaluated using Chi‐squared or Fisher's exact test. The event‐free survival curve was plotted using the Kaplan–Meier method, and their differences were assessed by the log‐rank test. A multivariate Cox hazards regression model was used to evaluate the hazard ratio (HR) for outcomes in terms of the 95% confidence interval (CI). Statistical significance was set at *p* < 0.05.

## 3 Results

### 3.1 Baseline Characteristics of the Study Cohort

**Figure 1:**
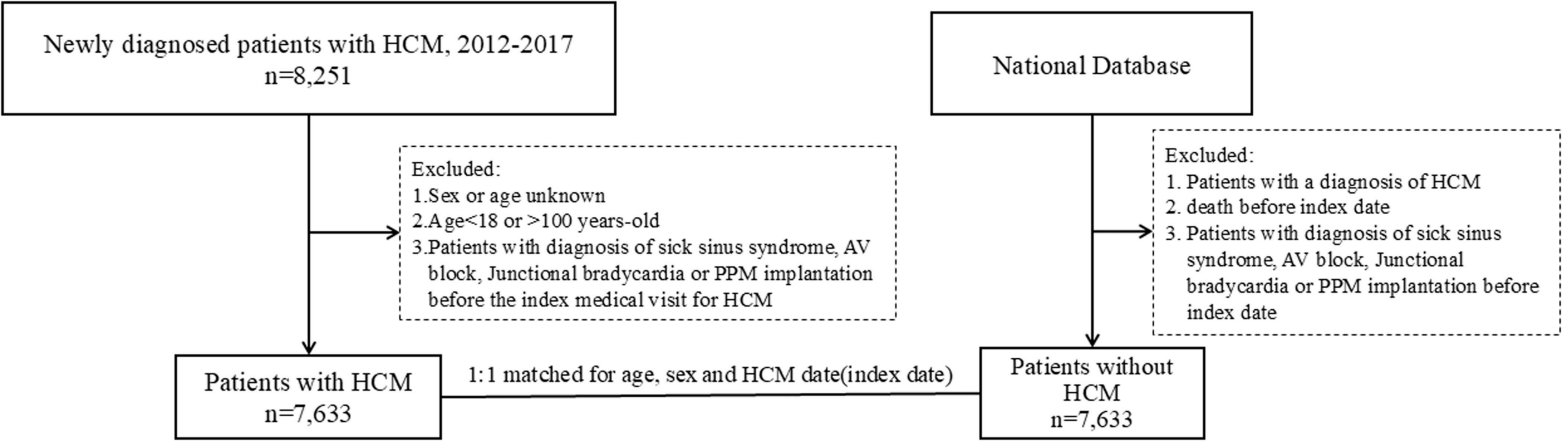
Flow chart of the study cohort. HCM, hypertrophic cardiomyopathy.

A total of 8251 patients with HCM were identified in this cohort (Figure 1). After applying the exclusion criteria, 7633 patients were included in the study cohort. A control cohort was constructed by matching patients without HCM to the study cohort by age and gender in a 1:1 ratio. The baseline characteristics of both cohorts are shown in Table 1.

Age (*p* = 0.776) and gender (*p* = 0.883) distributions were similar between the two groups. The HCM group exhibited higher prevalence rates of cardiovascular comorbidities, including AF (control: 3.1%; HCM: 14.1%, *p* < 0.001), HF (control: 5.5%; HCM: 22.3%, *p* < 0.001), hypertension (control: 48.9%; HCM: 65.4%, *p* < 0.001), vascular disease (control: 5.6%; HCM: 7.9%, *p* < 0.001), hyperlipidemia (control: 25.2%; HCM: 32%, *p* < 0.001), ischemic heart disease (control: 16%; HCM: 41.4%, *p* < 0.001), PVD (control: 2.2%; HCM: 2.7%, *p* = 0.024), VHD (control: 4.2%; HCM: 20.8%, *p* < 0.001), COPD (control: 9.5%; HCM: 13.5%, *p* < 0.001), and renal diseases (control: 7.6%; HCM: 9.8%, *p* < 0.001). There were no significant differences in the prevalence of stroke or transient ischemic attack (TIA) between these two groups (control: 13.4%; HCM: 13.1%, *p* = 0.703). The prevalence of diabetes mellitus in the HCM group was lower than that in the control group (control: 26.7%; HCM: 20.4%, *p* < 0.001).

Antihypertensive drugs, including beta‐blockers (control: 15.8%; HCM: 34.9%, *p* < 0.001) and angiotensin‐converting enzyme inhibitors (ACEIs) or angiotensin receptor blockers (ARBs) (control: 14.4%; HCM: 23.6%, *p* < 0.001), were more frequently used in the HCM group. Additionally, antithrombotic agent aspirin (control: 15.8%; HCM: 27.5%, *p* < 0.001) was more frequently used in the HCM group (Table 1). Only two patients (0.03% of the HCM cohort) underwent septal reduction therapy during the study period, including one patient who underwent septal myectomy and one who underwent alcohol septal ablation.

### 3.2 Bradyarrhythmia and PPM Implantation Risks in This Cohort

After a follow‐up duration of 4.05 ± 2.03 years, HCM patients had a high prevalence of sick sinus syndrome (control: 17 events, 0.2%; HCM: 73 events, 1.0%, *p* < 0.001), AV block (control: 16 events, 0.2%; HCM: 68 events, 0.9%, *p* < 0.001), and junctional bradycardia (control: 41 events, 0.5%; HCM: 116 events, 1.5%, *p* < 0.0001) compared to the control group. The incidence rate of bradyarrhythmic events was significantly higher in the HCM cohort (control: 74 events, 2.39/1000 patient‐years (PYs); HCM: 257 events, 8.47/1000 PYs, *p* < 0.001). Additionally, the incidence rate of PPM implantation was higher in the HCM group (control: 31 events, 0.99/1000 PYs; HCM: 248 events, 8.04/1000 PYs, *p* < 0.001).

Table 2 shows the risk factors associated with bradyarrhythmia and PPM implantation. In this cohort, the presence of advanced age (HR: 1.03, *p* < 0.001), HCM (HR: 2.15, *p* < 0.001), AF (HR: 1.71, *p* < 0.001), ischemic heart disease (HR: 1.44, *p* < 0.001), VHD (HR: 1.40, *p* = 0.003), and renal diseases (HR: 1.43, *p* = 0.008) were associated with increased risks of bradycardic events. Compared to the controls, the presence of advanced age (HR: 1.01, *p* = 0.033), HCM (HR: 6.58, *p* < 0.001), AF (HR: 2.17, *p* < 0.001), and HF (HR: 1.48, *p* = 0.007) were associated with higher risks of PPM implantation. The presence of hypertension (HR: 0.77, *p* = 0.042) was associated with lower risks of PPM implantation.

**Table 2:** Risk factors for bradyarrhythmia and pacemaker implantation in this cohort.

Variable	Bradyarrhythmia			PPM implantation		
	Adjusted HR	95% CI	*p* value	Adjusted HR	95% CI	*p* value
Age, years	1.03	(1.02−1.04)	< 0.0001	1.01	(1.00−1.02)	0.033
Gender						
Male	1.00	─	─	1.00	─	─
Female	0.98	(0.82−1.19)	0.865	0.80	(0.62−1.03)	0.079
HCM	2.15	(1.73−2.67)	< 0.0001	6.58	(4.45−9.75)	< 0.0001
Diabetes mellitus	1.15	(0.93−1.42)	0.198	0.90	(0.65−1.23)	0.490
Atrial fibrillation	1.71	(1.35−2.16)	< 0.0001	2.17	(1.63−2.89)	< 0.0001
Heart failure	1.04	(0.83−1.31)	0.739	1.48	(1.12−1.96)	0.007
Hypertension	0.92	(0.75−1.12)	0.409	0.77	(0.59−0.99)	0.042
Stroke or transient ischemic attack	1.00	(0.78−1.28)	0.999	1.36	(0.99−1.87)	0.061
Vascular disease	0.74	(0.49−1.10)	0.134	0.84	(0.49−1.43)	0.519
Hyperlipidemia	0.94	(0.77−1.15)	0.537	1.03	(0.78−1.35)	0.835
Ischemic heart disease	1.44	(1.19−1.76)	0.000	1.17	(0.90−1.52)	0.237
Peripheral vascular disease	1.06	(0.55−2.04)	0.866	1.36	(0.58−3.19)	0.480
Valvular heart disease	1.40	(1.13−1.74)	0.003	1.18	(0.89−1.58)	0.256
Chronic obstructive pulmonary disease	0.90	(0.69−1.17)	0.436	1.04	(0.74−1.47)	0.813
Renal disease	1.43	(1.10−1.86)	0.008	1.07	(0.72−1.61)	0.733
Beta blocker	1.20	(0.98−1.46)	0.074	0.96	(0.73−1.24)	0.734
ACEI or ARB	1.09	(0.88−1.35)	0.425	0.98	(0.73−1.31)	0.895
ASA (aspirin)	1.06	(0.87−1.31)	0.555	0.96	(0.72−1.26)	0.744

*Note:* HR was adjusted for all variables in this table. Bradyarrhythmia includes sick sinus syndrome, AV block, and junctional bradycardia.

Abbreviations: ACEI, angiotensin‐converting enzyme inhibitors; ARB, angiotensin II receptor blockers; HCM, hypertrophic cardiomyopathy; HR, hazard ratio.

**Figure 2:**
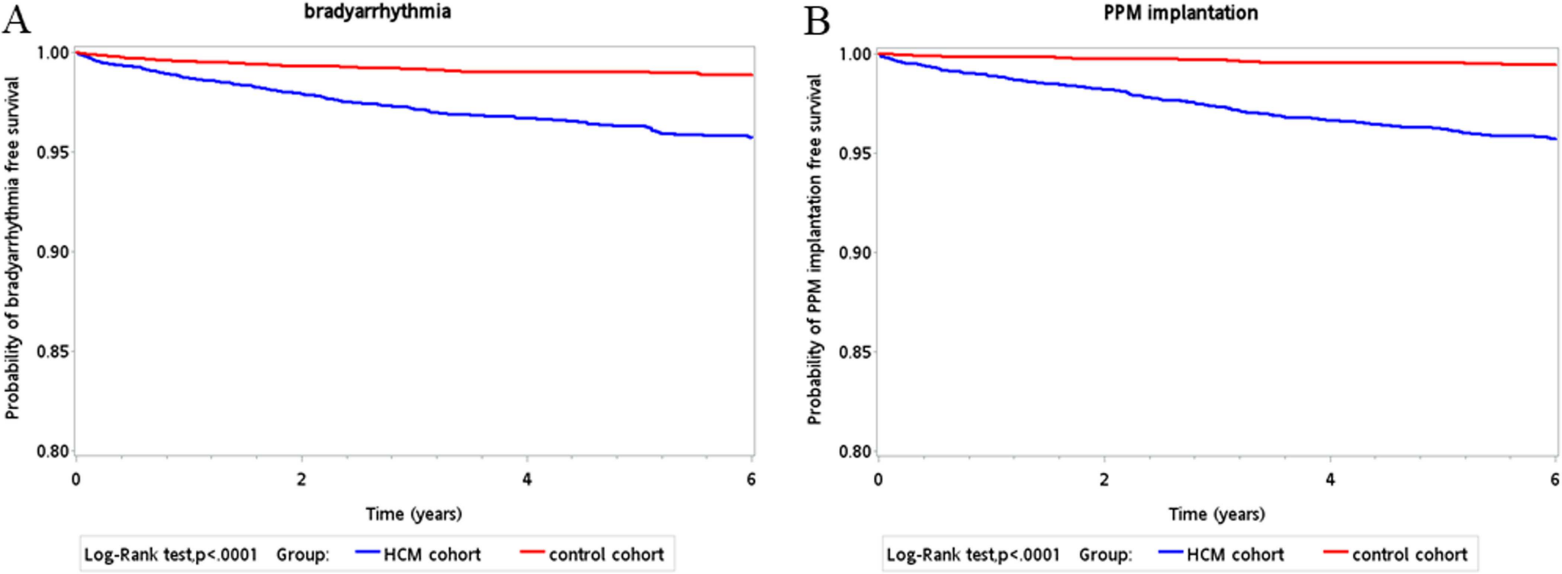
Kaplan–Meier event‐free survival curves for (A) bradyarrhythmia and (B) permanent pacemaker (PPM) implantation events in patients with/without hypertrophic cardiomyopathy (HCM).

Figure 2 shows the event‐free survival curves for bradyarrhythmia (Figure 2A) and pacemaker implantation events (Figure 2B) in patients with and without HCM. Patients with HCM had significantly higher event rates than controls for both outcomes (*p* < 0.001 for both).

### 3.3 Risk Factors for Bradyarrhythmia or PPM Implantation in HCM Patients

Table 3 shows the risk factors associated with bradyarrhythmia or PPM implantation in patients with HCM. In HCM patients, the presence of advanced age (HR: 1.03, *p* < 0.001), AF (HR: 1.61, *p* < 0.0001), ischemic heart disease (HR: 1.36, *p* = 0.006), and VHD (HR: 1.30, *p* = 0.032) were associated with increased risks of bradyarrhythmia, while hypertension (HR: 0.74, *p* = 0.007) was correlated with decreased risk. HCM patients with AF (HR: 2.12, *p* < 0.0001) and HF (HR: 1.61, *p* = 0.001) exhibited a higher risk of PPM implantation, while those with female gender (HR: 0.74, *p* = 0.033) and hypertension (HR: 0.67, *p* = 0.003) had lower risk.

**Table 3:** Clinical characteristics for bradyarrhythmia and pacemaker implantation risks in HCM patients.

Variable	Bradyarrhythmia			PPM implantation		
	Adjusted HR	95% CI	*p* value	Adjusted HR	95% CI	*p* value
Age, years	1.03	(1.02–1.04)	< 0.0001	1.01	(1.00–1.02)	0.199
Gender						
Male	1.00	─	─	1.00	─	─
Female	0.99	(0.79–1.23)	0.916	0.74	(0.57–0.98)	0.033
Diabetes mellitus	1.21	(0.93–1.56)	0.153	0.88	(0.62–1.25)	0.474
Atrial fibrillation	1.61	(1.24–2.08)	0.000	2.12	(1.58–2.84)	< 0.0001
Heart failure	1.04	(0.81–1.34)	0.742	1.61	(1.21–2.15)	0.001
Hypertension	0.74	(0.59–0.92)	0.007	0.67	(0.51–0.88)	0.003
Stroke or transient ischemic attack	0.94	(0.69–1.27)	0.678	1.37	(0.97–1.93)	0.079
Vascular disease	0.74	(0.46–1.19)	0.209	0.85	(0.48–1.51)	0.583
Hyperlipidemia	0.90	(0.71–1.15)	0.408	1.03	(0.77–1.38)	0.826
Ischemic heart disease	1.36	(1.10–1.70)	0.006	1.08	(0.82–1.41)	0.605
Peripheral vascular disease	1.43	(0.70–2.93)	0.325	1.66	(0.69–3.96)	0.256
Valvular heart disease	1.30	(1.02–1.64)	0.032	1.15	(0.85–1.55)	0.367
Chronic obstructive pulmonary disease	0.87	(0.64–1.18)	0.370	1.04	(0.72–1.50)	0.828
Renal disease	1.10	(0.78–1.55)	0.570	0.85	(0.53–1.37)	0.505
Beta blocker	1.10	(1.88–1.37)	0.428	0.94	(0.72–1.24)	0.670
ACEI or ARB	1.03	(0.80–1.31)	0.839	1.03	(0.76–1.39)	0.843
ASA (Aspirin)	1.10	(0.87–1.38)	0.443	1.01	(0.76–1.34)	0.954

*Note:* HR was adjusted for all variables in this table. Bradyarrhythmia includes sick sinus syndrome, AV block and junctional bradycardia.

Abbreviations: ACEI, angiotensin‐converting enzyme inhibitors; ARB, angiotensin II receptor blockers; HCM, hypertrophic cardiomyopathy; HR, hazard ratio.

## 4 Discussion

This study has three main findings: (1) Patients with HCM had higher risks of bradyarrhythmia and PPM implantation compared to the controls; (2) HCM patients with concurrent AF exhibited a higher risk of bradyarrhythmia and PPM implantation; and (3) HCM patients with HF had an increased risk of PPM implantation.

### 4.1 HCM and Risk of Bradyarrhythmia and PPM Implantation

HCM is an autosomal‐dominant disease caused by mutations in sarcomere genes such as β‐myosin heavy chain and α‐tropomyosin, leading to myocyte disarray, hypertrophy, ischemia, and fibrosis [7]. Although ventricular tachyarrhythmias are widely recognized as an important cause of sudden cardiac death in HCM patients, bradyarrhythmia can also substantially affect clinical outcomes; however, the risks of bradyarrhythmic events and PPM implantation are infrequently reported in HCM patients [1, 4, 5, 6]. In a cohort of 161 patients with HCM, bradyarrhythmic events occurred in 8% (13 patients) during a follow‐up period of 5.5 ± 4.4 years [10]. In another cohort of 451 patients with HCM, primary bradyarrhythmia was the reason for PPM implantation in 36 patients (8%) [9]. These studies typically analyzed a limited number of HCM patients, and they also had no control groups for comparison, that is, the relative risk could not be determined. In the present study, which had a large number of HCM patients (*n* = 7633), 3.4% experienced a bradyarrhythmic event, and 3.2% received a pacemaker implantation. These findings indicate that HCM is consistently associated with increased risks of bradyarrhythmic events and PPM implantation. Further prospective studies are needed to verify these findings.

In patients with HCM, the imbalance between oxygen supply and increased myocardial demand worsens with subaortic obstruction, mitral regurgitation, and diastolic dysfunction [8]. Fibrosis and ischemia may disrupt the cardiac conduction system, predisposing patients to both tachyarrhythmias and bradyarrhythmias. In a study of 155 HCM patients, 66% had prolonged sinoatrial conduction times, and 7% showed delayed sinus node recovery [11]. Additionally, repolarization dispersion from gap junction abnormalities and enhanced late sodium current contribute to conduction disturbances [12]. These findings suggest HCM affects not only the sinus node but the entire conduction system. In this study, we focused on bradyarrhythmic events, including sinus node dysfunction, AV block, and junctional bradycardia, which align with the known pathophysiological mechanisms of HCM.

### 4.2 AF and the Risk of Bradyarrhythmia and PPM Implantation in HCM Patients

AF is a common complication in HCM, affecting approximately 20%–25% of patients during the clinical course of the disease [8, 13, 14]. In patients with HCM, paroxysmal AF showed a higher burden of left atrial fibrosis and was associated with reduced atrial function and increased likelihood of AF. AF was also shown to be strongly associated with sinus node dysfunction, which was the leading indication for PPM implantation [15]. The interplay between sinus node dysfunction and AF remains incompletely understood, but appears to involve age‐related degenerative changes affecting not only the sinus node but also the atrial myocardium and the cardiac conduction system [16]. These changes promote atrial ectopy and dispersion of refractoriness, which predispose to AF. In turn, AF can lead to both electrical and structural remodeling of the sinus node, worsening sinus node function and reinforcing the cycle of bradyarrhythmia and AF [17].

In a study enrolling 169 patients with HCM who were eligible for an implantable cardioverter‐defibrillator (ICD), 11% of them developed bradyarrhythmia requiring pacing, with a higher prevalence observed in females and those with AF or on an anti‐arrhythmic drug [18]. In our cohort of HCM patients, AF per se was associated with an increased risk of bradyarrhythmia, which was linked to an increased risk of PPM implantation, and paralleled the results of previous studies. These findings indicate that detecting AF in patients with HCM is crucial due to the high risk of complications such as bradycardia and the potential need for PPM implantation.

### 4.3 Risk Factors for Bradyarrhythmia and PPM Implantation in HCM

HF is associated with left ventricular diastolic dysfunction and left atrial remodeling, leading to atrial dysfunction and subsequent bradycardia, particularly in patients with HCM. HF also plays a crucial role in the development of AF through left ventricular dysfunction, which promotes structural and electrical remodeling of the atrium and contributes to bradycardia [19]. Genetic mutations associated with HCM, such as those involving the MYH7 gene, can predispose patients to both HF and bradycardia [20]. In this study, we observed that HF was consistently a risk factor for PPM implantation, highlighting the importance of early detection and appropriate treatment of HF in HCM patients to prevent bradycardia and the need for PPM implantation. Further large‐scale prospective studies are warranted to confirm these observations.

In this study, ischemic heart disease was associated with increased risk of bradycardia in patients with HCM. Several possible reasons might account for this. First, myocardial ischemia may further aggravate damage to the cardiac conduction system, particularly in individuals with HCM [8]. Second, ischemic heart disease is a recognized risk factor for the development of AF in HCM patients, which can lead to bradycardia [15].

Interestingly, hypertension was associated with a lower risk of bradyarrhythmia and PPM implantation in patients with HCM. The reason for this association remains unclear. One possible explanation is that hypertensive patients are more likely to receive guideline‐directed medical therapies. However, the observed association may also reflect healthy‐adherer bias rather than a true protective effect of antihypertensive medications. Further studies are needed to elucidate their underlying mechanisms.

### 4.4 Clinical Implication

Although tachyarrhythmias such as ventricular fibrillation and ventricular tachycardia are well‐known causes of SCD in patients with HCM, bradyarrhythmia also poses an important but often under‐recognized risk. Clinicians should therefore assess not only ventricular arrhythmias but also bradyarrhythmic events, which may likewise contribute to SCD. AF is common in HCM and plays a significant role in the development of bradycardia, frequently leading to the need for pacemaker implantation.

Early detection of AF through ECG, Holter monitoring, or implantable loop recorders, followed by optimized management, is essential to improve outcomes. Comprehensive risk stratification, especially in HCM patients with coexisting AF, HF, or ischemic heart disease, is crucial for guiding individualized therapeutic strategies. Identifying risk factors and initiating appropriate treatment can help reduce the progression to clinically significant bradyarrhythmia and the subsequent requirement for PPM therapy.

### 4.5 Limitations

This study has several limitations. First, as an observational cohort study, causality could not be established. However, the large sample size of HCM patients strengthens the robustness of these findings. The mean follow‐up duration was relatively short (4.05 ± 2.03 years), which may have underestimated the long‐term incidence of bradyarrhythmia and PPM implantation in patients with HCM. Second, the diagnoses of HCM and primary outcomes were based on ICD‐9‐CM and ICD‐10‐CM codes, which may be subject to misclassification. Nonetheless, these diagnoses were made by physicians and coded by specialists, minimizing the likelihood of miscoding [21]. Third, detailed echocardiographic and ECG data were unavailable in the NHI database, precluding the assessment of key parameters, including interventricular septal thickness, left ventricular outflow tract pressure gradients, bradycardia duration, and AF subtypes (i.e., paroxysmal, persistent, or permanent). Consequently, we were unable to evaluate whether the burden or subtype of AF was associated with differential risks of bradyarrhythmia or PPM implantation. HCM phenotypes (e.g., obstructive vs. non‐obstructive or septal vs. apical) could also not be distinguished, and phenotype‐specific analyses were not feasible. Because these phenotypes may have distinct arrhythmic substrates and different risks of conduction abnormalities, bradyarrhythmia, and PPM implantation, future studies are warranted to clarify their impact. Fourth, among patients with HCM, only two patients (0.03% of the HCM cohort) underwent septal reduction therapy during the study period, including one septal myectomy and one alcohol septal ablation. Therefore, procedure‐related conduction disease was unlikely to have materially influenced our findings. Finally, while VHD was associated with increased risks of bradyarrhythmia and PPM implantation, further analysis of the mixed ICD codes for various valvular disease categories was not conducted.

## 5 Conclusions

Patients with HCM have significantly higher risks of bradyarrhythmia and PPM implantation compared to controls. HCM patients should be carefully monitored for the presence of AF and HF since they might increase the risks of future bradyarrhythmia and PPM implantation.

## Conflicts of Interest

The authors declare no conflicts of interest.

## Supporting information


Supporting File

## Data Availability

The data that support the findings of this study are available on request from the corresponding author. The data are not publicly available due to privacy or ethical restrictions.
