# Supplementary material for: Hypertrophic Cardiomyopathy and the Risk of Bradyarrhythmia and Permanent Pacemaker Implantation: A Nationwide Cohort Study
Source: Clin Cardiol. 2026 Aug 25;49(8):e70454. doi: 10.1002/clc.70454 (PMC13507695; doi:10.1002/clc.70454)
Supplement: Supplementary file 1 [file CLC-49-e70454-s001.docx]

**Supp. Table 1. Cardiovascular diseases and their corresponding ICD codes**

| **Diseases** | **ICD-9-CM codes** | **ICD-10-CM codes** |
| --- | --- | --- |
| Sick sinus syndrome | 427.81 | I49.5, R00.1 |
| Atrioventricular block | 426.0, 426.1 | I44.0, I44.1, I44.2, I44.39 |
| Junctional bradycardia | 427.89 | R00.1, I49.8 |
| Diabetes mellitus | 250, A181 | E08-E15 |
| Atrial fibrillation | 427.3 | I48, I49.0 |
| Heart failure | 428 | I50 |
| Hypertension | 401-405, A260, A269 | I10-I15, N26.2 |
| Stroke or transient ischemic attack | 430-438, A290-A294, A299 | G45-G46, I60-I69 |
| Vascular disease* | 410, 412, 440-445 | I21-I22,I70-I79 |
| Hyperlipidemia | 272, A182 | E71.30, E75.21-E75.22, E75.24, E75.3, E77.0-E77.1, E77.8-E77.9, E78.0-E78.9 |
| Coronary artery disease | 410-414, A270, A279, | I20-I25 |
| Peripheral vascular disease | 093.0, 437.3, 440, 441, 443.1, 443.8-443.9, 557.1, 557.9 | A52.01,I67.0,I67.1,I70,I71,I75,I77.819,I79.0,I73.1,I73.8,I73.9,I791,I79.8 |
| Valvular heart disease | 093.2, 394-397, 424, 746.3-746.6 | A52.01, I07-I09, I34-I38, Q22-Q23.3 |
| Chronic obstructive pulmonary disease | 490-493, 496 | J40-J45 |
| Renal disease | 403.01, 403.11, 403.91, 404.02, 404.03, 404.12, 404.13, 404.92, 404.93, 582, 583.0-583.7, 585, 586, 588.0 | I12.0, I13.11, I13.2, N03.2, N06.2- N06.5, N07.2- N07.5, N18-N19, N25 |

ICD-10-CM: International Classification of Diseases, 10^th^ Revision, Clinical Modification.

*Defined as myocardial infarction or peripheral vascular disease
